# Real-Time Estimation of the Short-Run Impact of COVID-19 on Economic Activity Using Electricity Market Data

**DOI:** 10.1007/s10640-020-00467-4

**Published:** 2020-08-04

**Authors:** Carlo Fezzi, Valeria Fanghella

**Affiliations:** 1 Department of Economics and Management, University of Trento, Via Vigilio Inama 5, 38122 Trento, Italy; 2 Land, Environment, Economics and Policy Institute (LEEP), Department of Economics, University of Exeter Business School, Exeter, UK

**Keywords:** COVID-19, Coronavirus, Economic impacts, Lockdown, GDP, Electricity quantity, Wholesale electricity markets, Pandemic, Fixed-effect regression, High-frequency estimates, Real-time monitoring

## Abstract

In response to the COVID-19 emergency, many countries have introduced a series of social-distancing measures including lockdowns and businesses’ shutdowns, in an attempt to curb the spread of the infection. Accordingly, the pandemic has been generating unprecedented disruption on practically every aspect of society. This paper demonstrates that high-frequency electricity market data can be used to estimate the causal, short-run impacts of COVID-19 on the economy, providing information that is essential for shaping future lockdown policy. Unlike official statistics, which are published with a delay of a few months, our approach permits almost real-time monitoring of the economic impact of the containment policies and the financial stimuli introduced to address the crisis. We illustrate our methodology using daily data for the Italian day-ahead power market. We estimate that the 3 weeks of most severe lockdown reduced the corresponding Italian Gross Domestic Product (GDP) by roughly 30%. Such negative impacts are now progressively declining but, at the end of June 2020, GDP is still about 8.5% lower than it would have been without the outbreak.

**Electronic supplementary material:**

The online version of this article (10.1007/s10640-020-00467-4) contains supplementary material, which is available to authorized users.

## Introduction

COVID-19, the disease caused by Severe Acute Respiratory Syndrome Coronavirus 2 (SARS-COV-2), first struck in China’s Hubei Province at the end of 2019 (Zhu et al. 2020). It quickly spread across the globe and was recognized by the World Health Organization (WHO) as a pandemic on the 11th of March 2020 (WHO 2020). At the time of writing, at the beginning of July 2020, the pandemic has resulted in excess of 11 million confirmed cases and more than 500,000 deaths. In order to reduce the pace of the infection, most countries introduced a variety of containment strategies, such as lockdowns, international and national travel restrictions, social-distancing and shutdowns of non-essential businesses, schools and public offices. These measures have saved lives by reducing the contagion and alleviating the burden on health care systems (e.g. Anderson et al. 2020). However, they have also generated substantial disruption on practically every aspect of social and economic life.

Preliminary studies indicate that the reduction in economic activity has been extremely significant. According to the most recent estimates (Eurostat 2020a, b; Organization for Cooperation and Development (OECD) 2020), practically all developed economies have experienced a contraction in the first quarter of year 2020. Furthermore, the prospect of an international recession much deeper than the financial crisis of 2008–2009 is extremely likely (International Monetary Fund (IMF) 2020).

Albeit informative, official statistics have two main drawbacks. First, they are typically released with a delay of at least 3 months.^1^ Second, in the context of the present pandemic, they simply provide an overall picture of the status of the economy, but fail to disentangle the impact of COVID-19 from those of all other factors affecting production and consumption. In other words, they do not offer any causal estimates of the impact that the virus and the related containment measures are having on economic activities. However, in this time of uncertainty and economic downturn, policy makers urgently require timely indicators to (1) monitor in real-time the impact of COVID-19 on the economy and (2) understand the causal impact of the policies designed to respond to the COVID-19 pandemic, including both those implemented to contain the virus and those developed to stimulate production and consumption after restrictions are lifted.

This paper shows how high-frequency electricity market data can provide both types of information. Electricity is traded on hourly (or even half-hourly) bases in most developed countries across the world and up-to-date information on consumption is publicly accessible via the system operators’ websites. In developed countries, electricity contributes to virtually every human activity and the relationship between electricity consumption (often indicated by the term “load”) and economic development is well established in the energy economics literature (e.g. Kraft and Kraft 1978; Chen et al. 2007; Stern 2018). By taking advantage of this strong link, satellite data on night lights are used to provide geographically disaggregated GDP estimates (Henderson et al. 2012) and even to forecast economic growth (Galimberti 2020). The relationship between electricity consumption and economic activities is particularly strong in the short-run. For example, significant drops in load occur during nighttime, weekends and public holidays when many businesses are shut down, creating the characteristic multi-level (daily, weekly and annual) seasonality of electricity markets’ time-series (Bunn and Farmer 1985). This paper develops a methodology to harness the wealth of information contained in electricity market data in order to monitor in real-time the state of the economy and to estimate the causal impact of the pandemic.

We illustrate our approach using daily data from the Italian day-ahead power market. Italy was the first European country to experience the outbreak of COVID-19 and, therefore, the one with the longest history of coping with the virus. It was also the first European country to implement a series of nation-wide restrictions on social and economic activities in an attempt to reduce the spread of the pandemic. Since the Italian Government introduced (and then lifted) a variety of measures of gradually increasing severity, our daily information allows us to separately estimate the short-run economic impact of each of the different restrictions. For all these reasons, Italy is a highly informative case-study regarding both the impact of containment strategies and the possible path of economic recovery. Taking into consideration the appropriate caveats, our estimates also provide valuable insights on how economic activities may react to future restrictions.

Our identification strategy relies on a fixed-effect estimator and on daily electricity load information for the years 2015–2020. We estimate the impact of COVID-19 on consumption and then apply a simple and yet effective rescaling approach in order to derive the implications for GDP. Our best estimate of the impact of the virus in the first quarter of 2020 in Italy is a 5.1% GDP reduction (with a 95% confidence interval between 3.8 and 6.4%), which is consistent with the official figures reporting a 5.3% drop (Eurostat 2020a, b; OECD 2020). Of course, our data is considerably more chronologically disaggregated and up-to-date and, therefore, we are not limited to the first quarter of 2020. On the contrary, we can distinguish the dynamic impacts of different policies and monitor the recovery of the economy thus far. We find that each newly introduced restrictive measure is followed by a significant reduction in economic activity. Not surprisingly, the strictest policies cause the largest economic impacts. For example, we estimate that the 3 weeks of most intense lockdown (with the temporary shutdown of a large number of factories) curtailed the corresponding GDP by about 30%. In similar fashion, when Italy started the gradual resumption of economic and social activities, our estimated impact gradually diminishes. However, at the end of June 2020 load has not yet reached the level that it would have been according to our counterfactual, signaling that the adverse impact of COVID-19 on the economy is still significant and ongoing.

## Electricity Market Data

The Italian electricity market opened in year 2004, which makes it one of the youngest power markets in Europe. Most Italian electricity consumption pertains to three large sectors: industry (39.6%), commercial and public services (32%) and residential (22.4%). The remaining 5.9% is allocated to transport, agriculture, forestry and fishing. The overall annual demand of about 300TWh is met by a mix of fossil fuels (about 65%), renewables (21%) and imports (14%).^2^

**Fig. 1:**
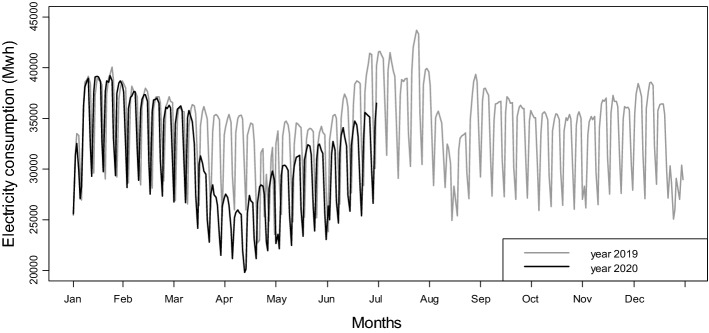
Daily average electricity consumption in years 2019 and 2020

We calculate daily electricity load from the publicity-available, hourly information provided by the Italian day-ahead electricity market system operator, Gestore dei Mercati Energetici (GME).^3^ Our dataset ranges from the 1st of January 2015 to the 30th of June 2020. To illustrate the key dynamics of electricity consumption and provide a first, visual inspection of the impact of COVID-19, Fig. 1 compares the daily load for years 2019 and 2020. Considering 2019, represented by the gray line, weekly seasonality is pronounced, with the reduced business activity in the weekends translating into roughly a 20% drop in load. Similar sharp falls also characterize public holidays. While these features are common to all electricity markets across the world, in Italy the effect of economic activities on load is also visible from the substantial reduction in consumption during the two central weeks of August, which is when the majority of Italian businesses shut down for the summer break. We also observe a smoother, annual seasonality, which follows the path of temperature, with peaks during winter and summer, when consumption for air-conditioning is at the highest.

Electricity consumption in 2020, shown by the black line, follows virtually the same path as in 2019 until the first week of March, when it diverges markedly. That is when the Italian Government started introducing a series of social-distancing measures in order to contain the escalating numbers of COVID-19 cases (a detailed timeline of the policies is reported in Table A1 in the Appendix in Electronic Supplementary Material). On the 10th of March the first national-level lockdown was implemented, quickly followed by the provisional closure of all shops, including bars and restaurants.^4^ The most restrictive measures were introduced in the last week of March and enforced until the second week of April. During this period, most factories were shut down and only supermarkets, pharmacies and other essential services were allowed to operate, but with reduced opening hours.^5^ Not surprisingly, this is when the gap between the electricity loads of the 2 years is at the widest. Production and consumption steadily recovered in the following weeks, with the gradual re-opening of the economy. However, at the end of June we can still observe a substantial gap between the 2 years.

**Fig. 2:**
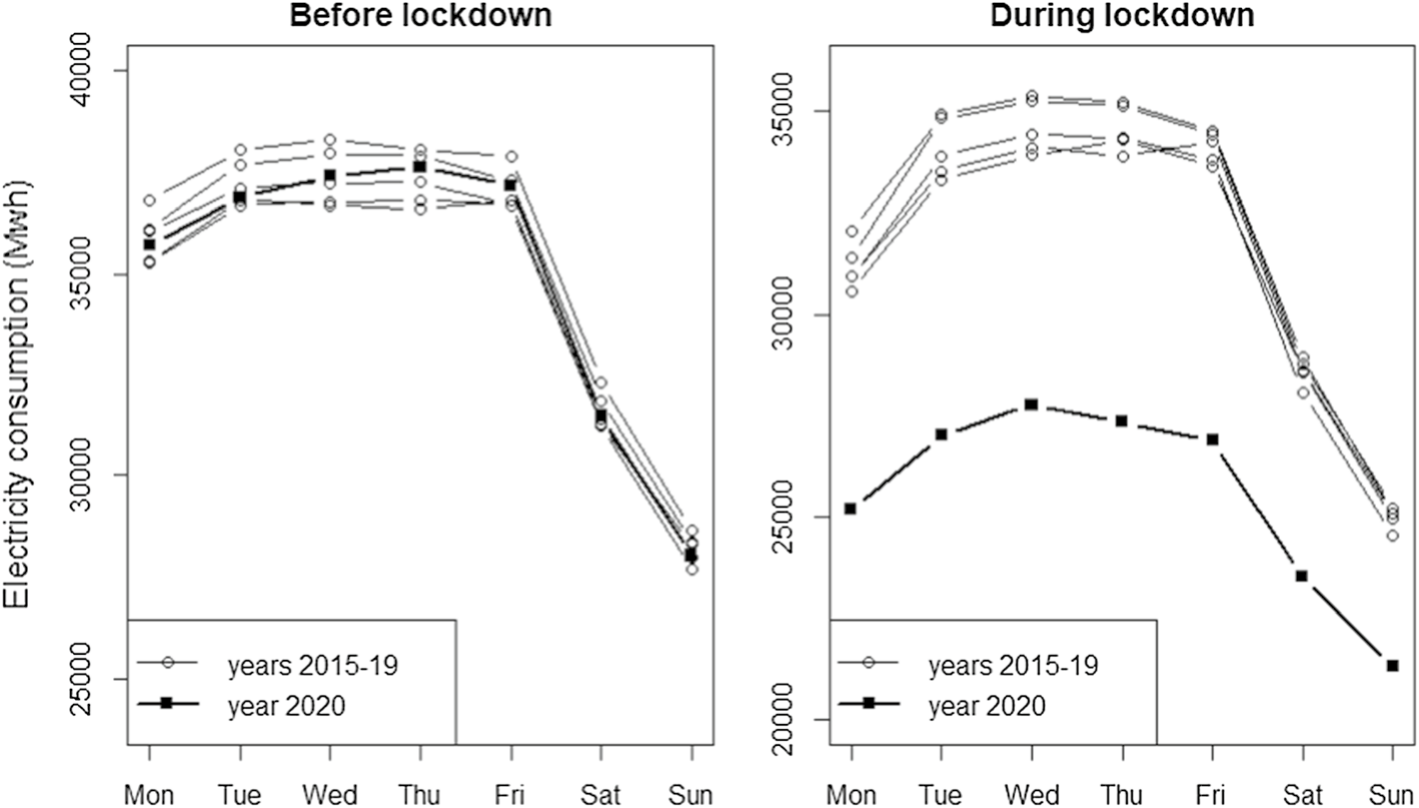
Average electricity consumption in each day of the week. *Notes:* For all years the “before lockdown” period consists of weeks 5–9 (35 days in February and March), while the “during lockdown” period consists of weeks 12–16 (35 days in March and April)

Figure 2 examines the impact of COVID-19 containment policies in more detail. It compares the weekly seasonality across all years in our 2015–2020 sample, distinguishing between two periods: (1) right before the 2020 lockdown dates (weeks 5–9 in February and March) and (2) during the most restrictive 2020 lockdown times (weeks 12–16 in March and April). Prior to the lockdown the pattern of electricity consumption in 2020 appears to be close to the average level of the previous 5 years, arguably a result of the slow GDP growth and of increased energy-efficiency (e.g. Malinauskaitea et al. 2019). However, during the lockdown weeks, the difference between 2020 and the previous years is remarkable. In that period, consumption in the weekdays of 2020 is comparable to that of the weekends of 2015–2019, while during the weekends of 2020 we observe an even lower load.

All these findings confirm the robust short-run relationship between the intensity of economic activities and electricity consumption. As mentioned earlier, another important determinant of load is air temperature. To represent this variable, we use an average of the mean daily temperature in the two largest Italian cities: Rome and Milan. We download this information up to the 13th of May 2020 from the University of Dayton archive.^6^

## Modelling Approach

The visual inspection of the data given in the previous section highlighted that electricity consumption in 2020 exhibited very similar patterns to those in the previous 5 years, at least up to the day in which the first containment measures were introduced. Therefore, the information in those earlier years can be used to construct a plausible counterfactual for what electricity consumption would have been in year 2020 in the absence of the pandemic. This line of reasoning has been already used to show that, in most countries, official figures greatly underestimate the real death toll of the outbreak (e.g., Ciminelli and Garcia-Mandicó 2020).

Indicating with *t* the daily steps of our time-series, our base model is:

1 $$y_{t} = \beta_{0} + \mathop \sum \limits_{j = 1}^{6} \beta_{j} d_{jt} + \mathop \sum \limits_{h = 1}^{2} \beta_{h} d_{ht} + \gamma_{w} + \gamma_{w,2020}^{*} + f\left( {temp_{t} } \right) + u_{t} ,$$

where *y*_*t*_ is the natural logarithm of electricity load, *d*_*jt*_ are six dummy variables identifying the day of the week (with Monday as the baseline), *d*_*ht*_ are two dummy variables identifying official public holidays and other observances, *γ*_*w*_ = are week-of-the-year fixed effects, *γ*_*w,*2020_* are week-of-the-year fixed effects interacted with a dummy variable identifying year 2020, *temp*_*t*_ = is the average air temperature and *f*(.) a non-linear functional form, *β*s are the remaining a parameters to be estimated and *u*_*t*_ is the random component.^7^

This specification is designed to capture all the peculiar features of electricity consumption and so isolate the causal impact of COVID-19. The week-of-the-year fixed effects *γ*_*w*_ encompass the slow-moving yearly seasonality of electricity load connected to both weather and cultural habits, such as the distinctive drop in consumption in the central 2 weeks of August that we mentioned in the previous section. The non-linear effect of the short-term variation in temperature is represented by *f*(.), which we specify as joint piecewise linear function as *δ*_*1*_*temp*_*t*_+ *δ*_*2*_(*temp*_*t*_ − *k*)*d*_*kt*_, where *k* is the threshold in which the relationship between temperature and load reverts and *δ*_*1*_, *δ*_*2*_ are the parameters to be estimated. This flexible specification generates a V-shaped function. We set *k* to 62 °F (about 16.5 °C) by visually inspecting the scatterplot of the data (see Figure A1 in the Appendix in Electronic Supplementary Material). Furthermore, the effects of the weekly seasonality and the public holiday effects are captured by the corresponding dummy variables.

Our model does not include electricity price. In fact, a peculiar characteristic of electricity markets is that the demand function can be considered as completely inelastic in the short-run, since the majority of final consumers do not purchase on the wholesale exchange but, rather, are supplied by utility companies at fixed tariffs (e.g., Fezzi and Bunn 2010). These companies operate on the day-ahead market, but are required to fulfill their orders to the final consumers and, therefore, cannot respond to price variation. Because of this feature, short-run electricity load forecasting models do not typically include price information (e.g. Taylor et al. 2006) and, practically, all short-run price forecasting methods treat quantity as exogenous (e.g. Weron 2014; Fezzi and Mosetti 2020). In line with this long-standing literature, therefore, we exclude price effects from our short-run analysis of electricity consumption.

The key-parameters for our study are the coefficients *γ*_*w,*2020_*, which measure the impact of COVID-19. These interaction effects capture the differences between each week of year 2020 and the average of the corresponding week in the previous 5 years which cannot be explained by any of the other observed factors. If our model is correctly specified, the *γ*_*w,*2020_* parameters corresponding to the weeks before the outbreak (i.e. during January and February) should be not significantly different from zero. Of course, we still include these parameters in our model because they serve as implicit in-time placebo tests for our modelling assumptions. On the other contrary, we expect to estimate highly significant and negative *γ*_*w,*2020_* parameters when the COVID-19 containment measures are introduced, i.e. roughly from the second week of March 2020.

If the error component *u*_*t*_ is independently and identically distributed (*iid*), our model can be consistently estimated with ordinary least squares (OLS). A possible concern with this estimator is that the *iid* assumption may not be satisfied, since the unobserved factors represented by the stochastic component may be autocorrelated. This situation can be generated by autocorrelated measurement errors. For example, the average temperature in Milan and Rome is unlikely to perfectly represent the weather profile of the entire country and, therefore, some remaining demand variation is undoubtedly present in the error term. Since weather shocks are typically autocorrelated, this missing variation is likely to generate autocorrelation in the residuals of our model. Possible omitted variables (e.g. special events or dynamic adjustments in residential and commercial load to temperature variations) are also plausible causes of residual autocorrelation. We investigate this issue through two distinct approaches. In the first one, we apply to the OLS covariance matrix the heteroscedasticity and autocorrelation consistent (HAC) correction proposed by Newey and West (1987). By setting the maximum lag for the correlation weights at seven we also attempt to capture any remaining weekly seasonality. In the second approach, we impose an autoregressive AR(1) specification for the random component (i.e. *u*_*t*_ = *ϕu*_*t*−1_) and estimate the resulting model with maximum likelihood. As a further check of the robustness of our findings, we re-estimate the base model after removing the piecewise function of temperature from the equation, in order to evaluate the susceptibly of our estimates to omitted variable bias. As shown in the next section, none of these alternative specifications alter our estimates of the effect of COVID-19 lockdown in any significant way. We run our analyses in *R* (R Development Core Team 2006), using the packages *lmtest* (Hothorn et al. 2019), *MASS* (Ripley et al. 2013), *nlme* (Pinheiro et al. 2017) and s*andwich* (Zeileis 2004).

If our model is correctly specified and passes the time-placebo test discussed above, the coefficients *γ*_*w,*2020_* from week 11 to week 22 can be interpreted as the causal impact of COVID-19 on electricity load. Therefore, we can estimate the impact of the pandemic and lockdown by comparing daily in-sample predictions obtained by (1) the full model and (2) a model in which such coefficients are set to zero. Indicating these two predictions (on the original scale of the variable) respectively with $\hat{Y}_{t}$ and $\hat{Y}_{t}^{*}$, we can write the percentage impact of COVID-19 on electricity load as:

2 $$l_{t} = 100\left( {\hat{Y}_{t} - \hat{Y}_{t}^{*} } \right)/ \hat{Y}_{t}^{*} ,$$

and derive appropriate confidence intervals via Monte Carlo simulations.^8^

Ideally, in order to translate electricity load reductions into GDP impacts we would want to employ detailed information, disaggregated by industry type, on changes in electricity consumption, value added and amenability to distance-working solutions. Unsurprisingly, this wealth of data is not available, particularly at the daily time-scale of our analysis. As a second-best solution, we employ some deliberately simple and intuitive assumptions to transform our estimates of electricity load changes into GDP impacts.

We assume that, in the short run and at the national level, GDP changes are proportional to the changes in electricity consumption by all productive sectors (i.e. all sectors but the residential one). In order to evaluate this claim, we run a back-of-the-envelope calculation on GDP and electricity consumption information for Italy for the years 1990–2018.^9^ Both variables are non-stationary and, therefore, we compute a correlation analysis on the first differences (in percentage) in order to avoid measuring a spurious relation. We estimate a correlation coefficient of 0.88, indicating an extremely strong linear covariation in the long-run. We believe this relationship to be even stronger in the short-run, justifying the simple assumption of a 1:1 relationship in our calculations (the plot is reported in Figure A2 in the Appendix in Electronic Supplementary Material).

In order to derive the impact of COVID-19 on the electricity consumption of the productive sectors we need to rescale our estimates, which are calculated on the total load. We compare two simple approaches. In the first one, we assume that residential consumption has remained unaffected by the restrictions and, therefore, all the reduction in electricity load due to the COVID-19 can be traced back to the other sectors. In the second one, we follow recent International Energy Agency’s estimates (IEA 2020) reporting that residential consumption has increased by 40% during the lockdown, and rescale our calculations accordingly. The percentage GDP impacts following these two methods can be written as:

3 $$GDP_{{ 1 {\text{t}}}} = l_{t} 100/\left( { 100{-}r} \right),\,{\text{and}}$$

4 $$GDP_{{ 2 {\text{t}}}} = l_{t} 100/\left( { 100{-} 1. 4r} \right),$$

where *r* represents the percentage of consumption of the residential sector, which in Italy corresponds to 22.4% according to the most up-to-date IEA estimates (footnote 3). While for transparency we report both measures, we believe that (4) should be the preferred estimate during the lockdown months (March and April) while (3) should be more accurate for the post-lockdown period, i.e. from the month of May onward. Although extremely simple, the next section shows that these assumptions provide results that are remarkably close to the official estimates of the GDP changes during the first quarter of 2020. Of course, our results extend well beyond the first quarter and provide an up-to-date assessment of the status of the economic disruption caused by the pandemic.

## Results

Table 1 presents the parameter estimates of our electricity load specifications. All three models provide an impressive fit to the data with R^2^ statistics higher than 0.92. In the first column we report our base model (1). All parameters have the anticipated signs and magnitudes. The temperature parameters estimate an asymmetric V-shaped relationship, with one degree higher than the threshold of 62 °F increasing electricity load roughly as much as three degrees below, reflecting the demand for cooling and heating respectively. A possible explanation for this asymmetric response is that, in Italy, a significant share of heating is generated directly by natural gas and, therefore, does not impact on electricity consumption. Day-of-the-week and public-holidays dummy variables indicate that consumption drops significantly during weekends and festivities. It is worth mentioning that directly interpreting these coefficients as semi-elasticities is incorrect since the derivative of a function with respect to a dichotomous variable does not exist. Nevertheless, semi-elasticities can be computed by a simple rescaling operation, which depends on both the sign and the magnitude of the coefficient (e.g. Halvorsen and Palmquist 1980). For example, the coefficient of Sundays is − 0.237. The appropriate decrease in load compared to Mondays (the baseline category) is 21.1% and not 23.7%.^10^

**Table 1:** Electricity load equation estimates

	Model 1 *Base*		Model 2 *No temperature*		Model 3 *Autocorrelation*	
Intercept	10.47***	*0.03*	10.38***	*0.07*	10.42***	*0.02*
Tue	4.04***	*0.29*	4.10***	*0.31*	4.09***	*0.18*
Wed	4.78***	*0.29*	4.85***	*0.31*	4.88***	*0.23*
Thu	4.65***	*0.29*	4.74***	*0.31*	4.78***	*0.25*
Fri	3.75***	*0.29*	3.71***	*0.31*	3.70***	*0.25*
Sat	− 11.91***	*0.29*	− 11.92***	*0.31*	− 11.85***	*0.23*
Sun	− 23.66***	*0.29*	− 23.66***	*0.31*	− 23.70***	*0.18*
d_holiday1_	− 21.35***	*0.50*	− 21.27***	*0.53*	− 18.59***	*0.33*
d_holiday2_	− 5.43***	*1.04*	− 5.47***	*1.11*	− 0.40	*0.68*
*Temp*	− 0.21***	*0.03*	–	–	− 0.08*	*0.03*
(*Temp*-62)d_62_	0.81***	*0.05*	–	–	0.37***	*0.06*
Week_1,2020_	0.97	*1.44*	1.40	*1.53*	1.76	*2.32*
week_2,2020_	2.13	*1.44*	3.08*	*1.53*	2.99	*2.47*
Week_3,2020_	2.32	*1.44*	2.28	*1.53*	2.48	*2.47*
Week_4,2020_	2.09	*1.44*	1.60	*1.53*	1.16	*2.47*
Week_5,2020_	0.93	*1.44*	− 0.06	*1.53*	0.49	*2.48*
Week_6,2020_	0.31	*1.44*	0.06	*1.53*	− 0.22	*2.48*
Week_7,2020_	0.10	*1.44*	− 0.47	*1.53*	− 0.18	*2.48*
Week_8,2020_	− 0.25	*1.44*	− 0.86	*1.53*	− 0.78	*2.48*
Week_9,2020_	− 1.19	*1.44*	− 1.60	*1.53*	− 1.52	*2.48*
Week_10,2020_	− 0.01	*1.44*	0.11	*1.53*	− 2.55	*2.48*
Week_11,2020_	− 8.15***	*1.44*	− 8.48***	*1.53*	− 9.45***	*2.48*
Week_12,2020_	− 18.70***	*1.44*	− 18.36***	*1.53*	− 16.32***	*2.48*
Week_13,2020_	− 23.84***	*1.45*	− 22.66***	*1.54*	− 21.95***	*2.48*
Week_14,2020_	− 22.52***	*1.44*	− 21.62***	*1.53*	− 21.44***	*2.48*
Week_15,2020_	− 25.57***	*1.46*	− 25.67***	*1.56*	− 24.26***	*2.48*
Week_16,2020_	− 18.31***	*1.44*	− 18.35***	*1.54*	− 16.25***	*2.48*
Week_17,2020_	− 10.83***	*1.44*	− 11.40***	*1.53*	− 14.33***	*2.48*
Week_18,2020_	− 11.38***	*1.44*	− 12.03***	*1.53*	− 11.04***	*2.48*
Week_19,2020_	− 10.85***	*1.44*	− 11.00***	*1.53*	− 11.40***	*2.49*
Week_20,2020_	− 9.09***	*1.45*	− 7.34***	*1.53*	− 8.45***	*2.49*
Week_21,2020_	− 7.26***	*1.45*	− 5.16***	*1.53*	− 7.18**	*2.53*
Week_22,2020_	− 7.68***	*1.44*	− 9.27***	*1.53*	− 8.19***	*2.83*
Week_23,2020_	− 5.03***	*1.45*	− 7.89***	*1.53*	− 6.58**	*2.48*
Week_24,2020_	− 4.24**	*1.46*	− 8.27***	*1.53*	− 6.38*	*2.49*
Week_25,2020_	− 5.71***	*1.45*	− 8.01***	*1.53*	− 6.53**	*2.50*
Week_26,2020_	− 6.68***	*1.44*	− 6.58***	*1.53*	− 7.33**	*2.62*
Weekly FE	YES		YES		YES	
*ϕ*	NO		NO		0.67	
R^2^	0.940		0.932		0.926	
Log-likelihood	3945.54		3815.62		4323.199	
AIC	− 7711.09		− 7455.25		− 8464.38	

All parameters except the intercept multiplied by 100 to improve readability. In italics we report parameters’ standard errors. Significance levels are *0.05, **0.01 and ***0.001. Model 1 and 2 estimated with OLS (HAC standard errors returned slightly smaller intervals and, therefore, we report the original OLS standard errors for conservative inference), while Model 3 is estimated with maximum likelihood. Variables d_holiday1_ and d_holiday2_ indicate dummy variables for (1) public holidays and (2) observances, while the *temp* terms represent the piecewise linear function of temperature, *ϕ* is the error-term autocorrelation parameter. All models include 52 weekly fixed effects (weekly FE). N = 1979

The focus of our analysis are the parameters *γ*_*w,*2020_*, i.e. the coefficients of the interaction terms between year 2020 and the weekly dummy variables, reported in the rows from week_1,2020_ to week_26,2020_. The parameters of the first 10 weeks are all non-significant, indicating that until the beginning of March there were no unobserved factors distinguishing the pattern of electricity load in year 2020 from the weekly averages of the previous 5 years. Therefore, our specification passes the in-time placebo test. We interpret this result as supporting the causal interpretation of our estimates. More specifically, the impacts estimated by the parameters *γ*_*w,*2020_* from the 11th week onwards can be traced back to COVID-19 and not to any unobserved factors. All these parameters are negatively signed and strongly significant, in line with our expectations that the containment policies caused significant reductions in electricity load. The strongest reductions happened during the 3 weeks of most intense restrictions (weeks 13–15), where we estimate a causal effect of about – 20% on electricity load.

In the second column, we report the specification in which we removed temperature in order to examine if our results are affected by the omission of relevant explanatory variables. The coefficient of the 2nd week of year 2020 is now significant at the 5% level, signaling that this model does not capture some of the distinctive features of electricity consumption during the current year and, therefore, fails the in-time placebo test. Here the reason is obviously the omitted temperature: the 2nd week of 2020 was, in fact, markedly colder than the average of the previous 5 years and the corresponding dummy variable is capturing this discrepancy. Incidentally, this result confirms that our in-time placebo test is powerful enough to detect unobserved factors. Despite the temperature omission, the *γ*_*w,*2020_* coefficients measuring the impact of COVID-19 (i.e. weeks 11–26) remain remarkably stable and, therefore, our results are essentially unaffected by this misspecification.

The third column reports the maximum likelihood estimates of the model with an AR(1) error term. In this specification the R^2^ cannot be directly compared with those of the previous regressions, since this index does not take into account the parameter of random component. Instead, we can compare the Akaike’s Information Criteria and the log-likelihood. Both values clearly indicate that the performance of Model 3 is superior to the two alternatives. The *ϕ* coefficient is about 0.67, signaling substantial autocorrelation in the error component. The standard errors of this model are somewhat larger than those of the previous specification. This feature suggests that OLS (and also the HAC standard errors) slightly underestimate the uncertainty of our findings. Apart from this difference, all the parameters appear to be consistent with those of the simpler specifications.

Table 2 summarizes the estimated impact of the lockdown and social-distancing measures on electricity load, calculated as illustrated by Eq. (2), and on GDP, calculated using both the approaches described by Eqs. (3) and (4). In order to preserve space, we report only the results according to our best-fitting specification (Model 3). In bold we indicate what we believe is the preferred method to calculate GDP impacts for each month, taking into account that residential electricity consumption increased during the lockdown (IEA 2020) but should have reverted to its usual level when the restrictions limiting people’s movements were lifted (i.e. at the beginning of May 2020). In March our best estimate concludes that the pandemic caused a 15.4% drop in GDP which, rescaled to the entire first quarter, leads to a 5.1% overall reduction, with the 95% confidence interval being from a 3.8% to a 6.4% fall in GDP. Our mean value is remarkably close to the recession of 5.3% projected by Eurostat (2020a, b) and the OECD (2020). However, it is worth mentioning that the interpretation of our results is different from those of the official statistics. They estimate the variation of GDP from the previous quarter, while our model isolates, *ceteris paribus*, the impact of COVID-19. Nevertheless, in our case-study the two measures are not likely to differ substantially given the sluggish growth of the Italian economy prior to the pandemic. Comparing results over time, the most affected month is April 2020, with a GDP reduction of roughly 25%. On the other hand, we start witnessing some signs of recovery in May, for which estimate an average reduction of 11%, and in June, in which we register a decrease of 8.5%.

**Table 2:** Estimated monthly impacts of COVID-19

	Electricity (%)	GDP_1_ (%)	GDP_2_ (%)
March	− 10.6 [− 7.9; − 13.2]	− 13.7 [− 10.2; − 17.0]	− **15.4 [**− **11.5; 19.3]**
April	− 16.8 [− 14.3; − 19.3]	− 21.7 [− 18.4; − 24.9]	− **24.5 [**− **20.8; 28.1]**
May	− 8.8 [− 6.1; − 11.4]	− **11.3 [**− **7.8;** − **14.6]**	− 12.8 [− 8.8; 16.6]
June	− 6.6 [− 3.5; − 9.4]	− **8.5 [**−**4.6;** − **12.1]**	− 9.6 [− 5.2; 13.7]

Results according to Model 3 in Table 1. *Electricity* indicates the estimated impact on electricity load, *GDP*_*1*_ indicate the impact on GDP assuming that residential consumption has not changed, while *GDP*_*2*_ indicates the impact on GDP assuming that residential consumption increased by 40%. The square brackets report 95% confidence intervals calculated via 5000 Monte Carlo simulations. In bold we highlight our preferred GDP-change estimates

**Fig. 3:**
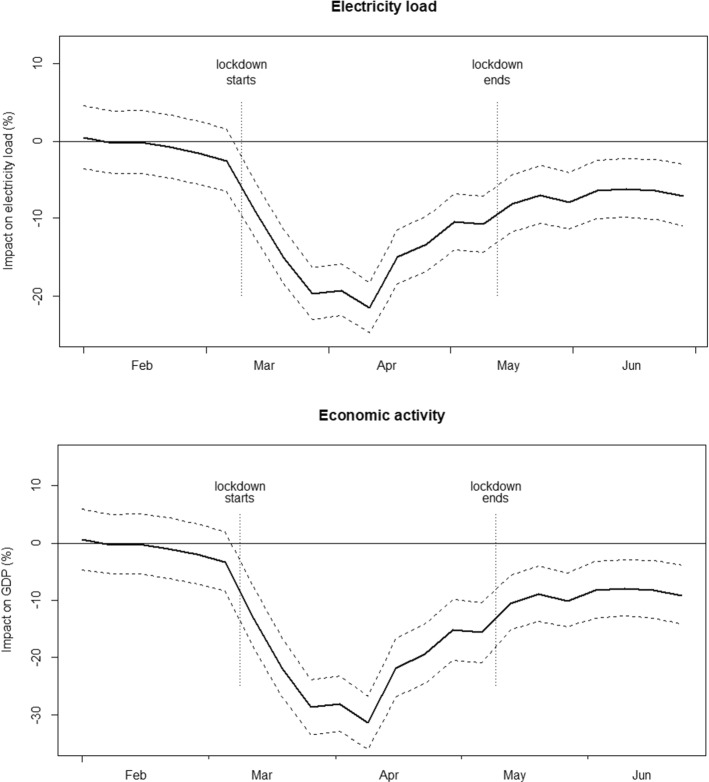
Estimated impact of COVID-19. *Notes*: The bold solid line is the estimated impact of COVID-19 on electricity load (top figure) and GDP (bottom figure) according to our preferred specification (Model 3) and the dashed line are the 95% confidence intervals calculated via 5000 Monte Carlo repetitions. GDP impacts are calculated according the Eq. (4) for the lockdown weeks and to Eq. (3) for all other periods

These dynamics are represented more in detail in Fig. 3. In line with our parameter estimates, impacts on both electricity load (top image) and GDP (bottom image) are non-significant for all the weeks before the outbreak. Starting from the introduction of the first lockdown measures in the second week of March 2020, each incremental constraint (once again, see Table A1 for the timeline of the lockdown policies in Italy) is followed by a significant drop in electricity consumption. In a similar fashion, the loosening of the restrictions, which started on the 3rd week of April 2020, prompted a resumption of economic activities and, therefore, our estimated negative impact steadily diminishes.

GDP dynamics follow roughly the same pattern but are somewhat amplified, with the strictest policies causing roughly a temporary 30% drop in GDP. Our method allows us to track impacts in real-time and, therefore, we report estimates until literally a few days before the final submission of this article, i.e. until the end of June 2020. To date, the Italian Government has lifted the strictest restrictions, but social-distancing requirements still constrain many economic activities. For example, shops and restaurants are open with reduced capacity, while the tourism sector is experiencing significant losses. Figure 3 indicates that the GDP recovery that started in the last week of April (and persisted in May) has, unfortunately, reached a *plateau* in June, in which we register a roughly constant 8.5% reduction compared to the counterfactual level. Therefore, at the time of writing we do not detect a full comeback of the economy, or even just a temporary rebound triggered by the rescheduling of the industrial processes halted by the 3-weeks shutdown in the months of March and April.

An important issue, which needs to be kept in mind when interpreting our most recent estimates, is international spillover effects. The production (Backus and Kehoe 1992), consumption (Cavaliere et al. 2008) and financial assets (Forbes and Rigobon 2002) of one country do not exist in isolation, but are inextricably connected to the economic conditions of the rest of the word. Since Italy was the first European country to introduce strict lockdown policies, we believe that our estimates correspond to the short-run causal impact of such early restrictions. However, by mid-April 2020 the crisis had spread across much of the world and, therefore, our estimates for the tail end of our data go beyond the impact of national policies and need to be interpreted by taking into account the global recession and the disruption of international supply-chains caused by the pandemic (e.g. Baldwin and Weder di Mauro 2020).

## Conclusions and Caveats

We develop a simple and practical methodology for estimating the short-term impact of COVID-19 on the economy by analyzing high-frequency electricity market data. The main advantage of our approach lies in its real-time nature. While official statistics (e.g. Eurostat 2020a, b; OECD 2020) are typically published with a delay of about 3 months, our method provides updated estimates of the economic impact of the pandemic on a weekly (or even daily) basis. In the current uncertain economic environment, timeliness is of essence for policy makers seeking to understand the current state of the economy and the impact of their policies. Our approach can be used to monitor in real-time the extent of the disruption caused by the pandemic. It can also be used to assess the effectiveness of the monetary and fiscal stimuli that many countries have introduced in order to address the crisis. A further strength of our empirical strategy is that it is widely applicable. It only requires data on electricity load and temperature, and such information is publicly available for virtually all developed economies in the world.

We illustrated our methodology using daily information from the day-ahead Italian electricity market. Italy was the first European country to both experience the outbreak of COVID-19 and to implement a series of nation-wide restrictions on social and economic activities in an attempt to curb the spread of the infection. Aggregating our weekly results (robust to different specifications and to in-time placebo testing), we estimate that these restrictions reduced Italian GDP by 5.1% during the 1st quarter of 2020, which is remarkably close to the preliminary figures published by Eurostat (2020a, b) and the OECD (2020). Of course, our approach produces estimates which are significantly more up-to-date and disaggregated. We estimate that the most severe impacts were registered during the 3 weeks of March and April 2020 when all non-necessary economic activities were required to halt production. Our preferred modelling specification estimates a 30% GDP reduction during this period. We also detect a gradual recovery right after, such that, by the end of June, economic activity was “only” 8.5% lower than what it would have been had the pandemic not occurred. This remaining recession is hardly surprising given that social-distancing requirements are still constraining economic activities, particularly in the cultural, catering and hospitality sectors. Our model can be routinely updated over time in order to monitor GDP dynamics and to track the status of the economy well ahead of the official statistics.

A number of comments and caveats need to be made when interpreting our findings. First, our analysis concerns only short-run effects. Over time the impacts of different national policies tend to overlap with each other, and spillover effects from other countries become gradually more important. Economic shocks are not limited by frontiers but, rather, reverberate across countries almost like a further form of contagion. These have consequences for both real (e.g. Backus and Kehoe 1992) and financial markets (Forbes and Rigobon 2002). This issue is likely to be negligible for our estimated GDP impacts in the initial weeks of restrictions, since Italy was the first country in Europe to grapple with the pandemic and implement lockdown policies. However, as the pandemic subsequently spread internationally, so our findings should progressively be interpreted as the general effect of the pandemic, rather than solely the response to a specific national policy. Despite these confounding factors and knock-on effects, the validity of our approach for monitoring the real-time status of the economy still stands.

Second, while we believe that at the national level postulating a 1:1 relationship between electricity load change (excluding residential users) and GDP change is satisfactory in the very short-run, our approach does not disentangle the heterogeneous impacts of the pandemic across sectors. Some activities, such as wholesale and retail food supply, as well as e-commerce, are likely to have experienced an increase in turnover, while small and medium companies in the manufacturing, hospitality and personal services have been the most adversely affected.^11^

Finally, our approach does not take into account the possibility of adaptation strategies such as a switch to home working. As companies become more adept at such modes of working, so the effect of future lockdown restrictions may become less severe. This consideration has to be balanced against the potential for long-term and repeated lockdowns exerting cumulative and progressively more adverse economic effects, including through the impacts on human wellbeing.

## Electronic supplementary material

Below is the link to the electronic supplementary material.

Supplementary material 1 (DOCX 83 kb)

## Footnotes

1 As an example, economic assessments for the fourth quarter of 2019 were available only in April 2020 for both the European Union (Eurostat 2020b) and the United States (Bureau of Economic Analysis, BEA 2020).

2 Source: International Energy Agency: https://www.iea.org/data-and-statistics.

3 GME historical data is available at: https://www.mercatoelettrico.org/En/Tools/Accessodati.aspx.

4 DPCM 9th of March, Official Gazette: https://www.gazzettaufficiale.it/eli/id/2020/03/09/20A01558/sg.

5 DPCM 22nd of March, Official Gazette: https://www.gazzettaufficiale.it/eli/id/2020/03/22/20A01807/sg and DPCM 25th of March, Official Gazette: https://www.gazzettaufficiale.it/eli/id/2020/03/25/20G00035/sg.

6 Source: http://academic.udayton.edu/kissock/http/Weather/default.htm. This website is not updated every day and, therefore, for the most recent month (June 2020) we used temperature information from the monitoring stations of the National Oceanic and Atmospheric Administration (https://www.ncdc.noaa.gov/cdo-web/).

7 Regarding the week-of-the-year fixed effects, we tested two alternative definitions. In the first one we used calendar weeks. With this approach, in different years the days covered by each fixed-effects do not correspond and the number of days represented by the first week can vary across years. In the second approach, we defined the fixed effects as groups of 7 days, always starting from the 1st of January of each year. Therefore, the only difference across years is caused by leap years. This second method provided a slightly superior fit and, therefore, is the one we adopt in the all our models.

8 Because of Jensen’s inequality (e.g. Silva and Tenreyro 2006), the predictions on the original variable’s scale are not simply the exponent of the predictions on the log, but actually depend on the distribution of the random component. The practical significance of this inequality is influenced by the magnitude of the residuals, i.e. by the noise-vs-signal ratio. In our specific case the difference is negligible since all our specifications present extremely high fit (all R^2^ are higher than 0.92) and, therefore, for simplicity we employ the Gaussian assumption, i.e. we use *Y*_t_ = exp{[log(*y*_t_)] + (*s*^2^/2)}, with *s* indicating the estimated standard deviation of the error component.

9 Source: IEA, https://www.iea.org/data-and-statistics and The World Bank, https://data.worldbank.org/.

10 Semi-elasticities *ξ* can be obtained using the following rescaling equation: *ξ *= 100(exp{*c*} – 1), where *c* indicates the coefficient of the dummy variable (Halvorsen and Palmquist 1980). This equation assumes that the coefficients is known, while in reality it is a random variable. The correct formula depends on the distribution of the error term (van Garderen and Shah 2002) but, in most cases, makes little difference, particularly when the parameters are highly significant. We tested different approximations but none changed our results in a meaningful way and, therefore, we opted for the above equation for simplicity.

11 OECD statistics: http://www.oecd.org/sdd/business-stats/statistical-insights-small-medium-and-vulnerable.htm.
