# Supplementary material for: Real-Time Estimation of the Short-Run Impact of COVID-19 on Economic Activity Using Electricity Market Data
Source: Environ Resour Econ (Dordr). 2020 Aug 4;76(4):885–900. doi: 10.1007/s10640-020-00467-4 (PMC7399598; doi:10.1007/s10640-020-00467-4)
Supplement: Supplementary file 1 — Supplementary material 1 (DOCX 83 kb) [file 10640_2020_467_MOESM1_ESM.docx]

**Appendix: Supplementary Information**

**Table A1:** COVID-19-containment measures’ timeline in Italy

| **Date** | **Measures** |
| --- | --- |
|  |  |
| *10/03/2020* | **Phase I begins**: the entire country is in lockdown |
| *12/03/2020* | *Lockdown tightening*: all shops (except supermarkets, pharmacies, stationers, gas stations), bars, restaurants, hair and nail salons are required to remain closed. |
| *14/03/2020* | Severe restrictions to domestic and international travels. Very significant reduction in transportation. |
| *23/03/2020* | *Lockdown tightening*: all unnecessary work activities a forced to shut down. Only supermarkets, pharmacies, and other essential services are allowed to operate. |
| *14/04/2020* | *Lockdown loosening*: some activities, such as bookshops, manufacturing and major infrastructure projects are allowed to re-open. |
| *27/04/2020* | Some regions with a low number of COVID-19 cases allow certain commercial activities to re-open and lift restrictions on regional mobility. |
| *04/05/2020* | *Lockdown ending*: the entire country enters **Phase II**. Commercial and production activities that can enforce the social-distancing protocol can re-open. Bars and restaurants can operate only for take-away service. Hairdressers are still closed. Restrictions to mobility across regions are lifted. |
| *18/05/2020* | **Phase II.bis**: Most regions allow bars, restaurants and hairdressers to re-open following strict social distancing protocols. Regions can autonomously decide which activities are allowed to re-open. |
| *03/06/2020* | Italy opens to international arrivals: residents of other European Union countries are allowed to visit to Italy for work and tourism with no quarantine period. |

**Figure A1**: Scatter-plot of air temperature and electricity consumption

**
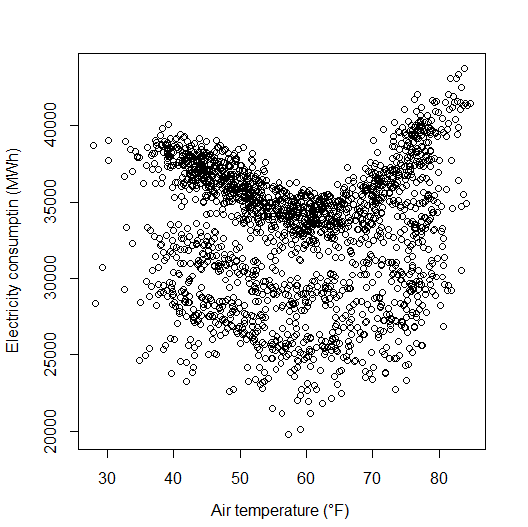
**

*Notes*: air temperature calculated as the daily average between Rome and Milan (time period: 01/01/2015 – 30/06/2020).

**Figure A2**: Scatterplot of yearly electricity consumption and GDP changes


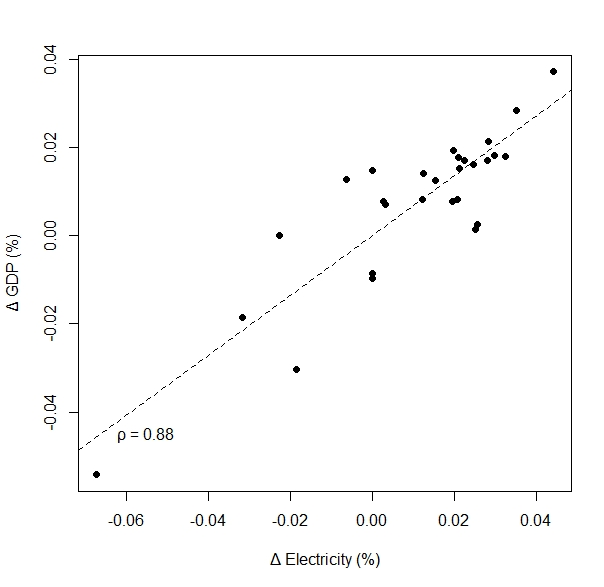


Notes: years 1990 to 2018, ρ indicates the correlation coefficient. GPD data from the World Bank (<https://data.worldbank.org/>), electricity load data from the IEA (<https://www.iea.org/data-and-statistics>).
